# Outcomes of surgical management of Ebstein anomaly and tricuspid valve dysplasia in critically ill neonates and infants

**DOI:** 10.1016/j.xjon.2023.08.007

**Published:** 2023-08-22

**Authors:** V. Reed LaSala, Edward Buratto, Halil Beqaj, Isabel Aguirre, Julian Maldonado, Nimrod Goldshtrom, Andrew Goldstone, Matan Setton, Ganga Krishnamurthy, Emile Bacha, David M. Kalfa

**Affiliations:** a Section of Pediatric and Congenital Cardiac Surgery, Division of Cardiac, Thoracic, and Vascular Surgery, New York-Presbyterian Morgan Stanley Children’s Hospital, Columbia University Medical Center, New York, NY; b College of Physicians and Surgeons, Columbia University, New York, NY; c Division of Neonatology, Department of Pediatrics, New York-Presbyterian Morgan Stanley Children’s Hospital, Columbia University Medical Center, New York, NY; d Division of Cardiology, Department of Pediatrics, New York-Presbyterian Morgan Stanley Children’s Hospital, Columbia University Medical Center, New York, NY

**Keywords:** congenital heart disease, Ebstein anomaly, tricuspid valve dysplasia, tricuspid valve, single ventricle, Starnes, cone

## Abstract

**Objective:**

To describe the surgical outcomes in neonates and infants who had surgery for Ebstein anomaly (EA) and tricuspid valve dysplasia (TVD).

**Methods:**

Retrospective chart review for all patients who underwent surgery for EA or TVD during the index hospitalization after birth at our institution from January 2005 to February 2023.

**Results:**

Fifteen symptomatic neonates and infants who had surgery for EA or TVD were included, 8 with EA and 7 with TVD. Eleven patients (73%) and 3 patients (20%) required preoperative inotropes and extracorporeal membrane oxygenation, respectively. Nine patients (60%) had a Starnes procedure and 6 patients (40%) had tricuspid valve repair (TVr). Mortality at last follow-up was 27% overall (n = 4/15), 22% after Starnes (n = 2/9) and 33% after TVr (n = 2/6), without a significant difference despite a greater-risk profile in the Starnes group. Postoperative day 1 lactate level was associated with mortality on Cox regression (hazard ratio, 1.45; *P* = .01). Three of 9 patients who had a Starnes procedure were or will be converted to a cone repair (1.5/2-ventricle repair).

**Conclusions:**

Mortality after surgery for EA or TVD during the index hospitalization after birth is still significant in the current era and is associated with a greater lactate level at postoperative day 1. The Starnes procedure and TVr had comparable outcomes despite a greater-risk profile in the Starnes group. An initial single-ventricle approach does not preclude conversion to biventricular or 1.5-ventricle repair.

## Graphical abstract

**Figure:**
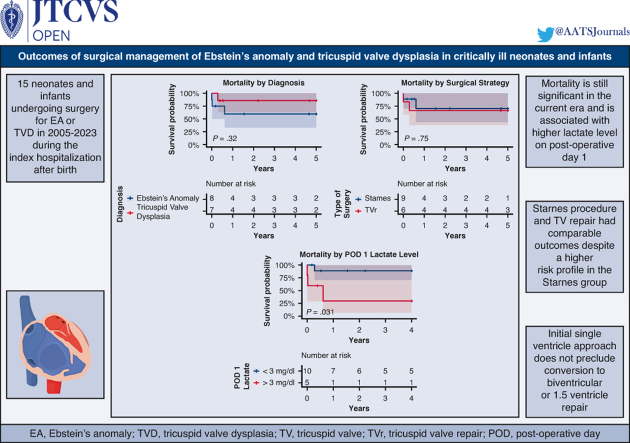

**Figure:**
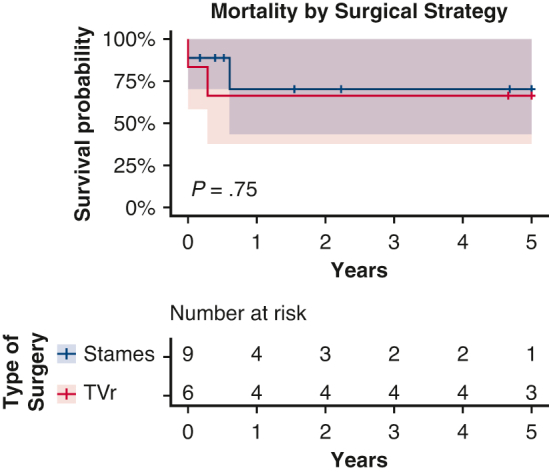
Kaplan–Meier plot of mortality by surgical strategy after neonatal surgery for EA and TVD.

**Central Message**

The Starnes procedure and TV repair had comparable outcomes despite a greater-risk profile in the Starnes group.

**Perspective**

Mortality after neonatal Starnes procedure or tricuspid valve repair for EA or TVD is associated with a greater lactate level at postoperative day 1. An initial single-ventricle approach does not preclude conversion to biventricular repair.

See Discussion on page 639.

Symptomatic neonatal and infant forms of Ebstein anomaly (EA) and tricuspid valve dysplasia (TVD) remain challenging clinical entities with an overall high mortality. These diseases present with a wide spectrum of clinical severity, often in the context of other anatomic defects. The most severe forms are characterized by severe tricuspid regurgitation (TR) and, in EA, a large atrialized, nonfunctional right ventricle (RV) resulting in right heart failure often progressing to circular shunting and severe acidosis, necessitating early surgical intervention.

Biventricular and univentricular surgical strategies can be offered to these challenging patients. Right ventricular exclusion (Starnes procedure) is advocated as a rapid and reproducible procedure but with the attendant drawbacks of single-ventricle physiology.^1^^,^^2^ Various tricuspid valve repair (TVr) techniques have been described to allow initial biventricular repair.^3^ Most recently, the cone procedure has been advocated as a more durable and effective technique for the repair of EA, yet it is a more complex operation that may prove time-consuming and technically challenging with fragile neonatal valves.^4^

Initial studies showed more favorable outcomes with the Starnes procedure,^5^ but as familiarity with TVr techniques has increased, equivalent overall outcomes have been demonstrated with biventricular repair.^6^ In particular, familiarity with the cone procedure has increased and this procedure can be successfully offered to infants.^7^^,^^8^ As yet, no consensus has been reached for the optimal approach to these challenging patients. In this study, we reviewed our experience with symptomatic neonatal and infant congenital tricuspid valve (TV) disease to describe outcomes of each surgical approach and risk factors for mortality.

## Methods

A retrospective chart review was performed for 29 consecutive patients who underwent surgery for EA or TVD within the first year of life at a single center between January 2005 and February 2023. Seven patients who underwent surgery primarily for the repair of an associated anomaly were excluded. Seven patients who were discharged home after the index hospitalization but then needed a TV surgery in the first year of life were also excluded in order to appropriately compare critically ill neonates and infants with EA or TVD. The remaining 15 patients who underwent cardiac surgery for symptomatic EA or TVD during the index hospitalization after birth were included. The data obtained from retrospective chart review of these 15 patients were analyzed. Operations were performed by 7 surgeons, who were each active during different segments of the 18-year study period. All echocardiograms were reviewed by attending cardiologists and diagnoses made based on the standard criteria. The primary outcome was mortality. Secondary outcomes were postoperative degree of TR in the patients who received TVr, need for reintervention on the TV, and ventricular status (single-ventricle, 1.5-ventricle, and 2-ventricle). Patients were grouped by ventricular pathway (2-ventricle vs single-ventricle vs 1.5-ventricle) based on the initial strategy (TVr/2-ventricle, Starnes/single-ventricle, Starnes with plan for future conversion) determined before performing the initial procedure. All patients who received the Starnes were and are currently being systematically evaluated for 1.5- or 2-ventricle conversion based on TV tissue quality, RV size and function, and the overall physiology and clinical status of the patient.

Functional pulmonary atresia (FPA) was defined by an anatomically patent right ventricular outflow tract without antegrade flow through the pulmonary valve. Circular shunt was defined by FPA with retrograde flow through the pulmonary valve, right-to-left flow across the atrial septal defect, and left-to-right flow across the patent ductus arteriosus. In this cohort, all patients with FPA had circular shunt physiology. The degree of TR was stratified into mild, moderate, and severe on the initial echocardiogram. A low cardiac output state/low systemic perfusion was defined by at least moderate systolic dysfunction on echocardiogram, preoperative inotropic support, extracorporeal membrane oxygenation (ECMO), or the presence of any end-organ dysfunction. Acute kidney injury was defined by a creatinine greater than 0.92 mg/dL for patients 0 to 14 days old and creatinine greater than 0.36 mg/dL for patients greater than 14 days old.^9^ Operative mortality was defined by death within 30 days of operation or death during the same hospitalization as the operation. Postoperative day 1 lactate was defined as the first lactate on the day after the operation.

The Starnes procedure consists of TV exclusion using a fenestrated patch, atrial septectomy, patent ductus arteriosus ligation, pulmonary artery ligation/division or banding, and Blalock–Taussing–Thomas shunt placement.^2^ Several techniques for TVr have been used, including annuloplasty, commissuroplasty, division of restrictive chordae, and cone repair.^3^^,^^4^

Statistical analysis was performed in R (R Foundation) and RStudio (Posit). Categorical variables were compared using the Fisher exact test, whereas continuous variables were compared using the Wilcoxon rank sum test. Time-dependent variables (ie, mortality) were analyzed using the Kaplan–Meier method, with groups compared using the log-rank test. Cox proportional hazards modeling was used to assess predictors of mortality. Median follow-up was 1.55 years (0.35-6.21 years). There were no patients lost to follow-up. This study was approved by the institutional review board (protocol number AAAU3640, approved October 3, 2022), and the need for consent was waived.

## Results

### Preoperative Characteristics Overall and Stratified by Diagnosis

Fifteen patients were identified who required surgery for EA or TVD during their index hospitalization after birth. Demographic information is summarized in Table 1. Median and interquartile range (IQR) for gestational age was 37 weeks (36-39 weeks). Median (IQR) birth weight was 2.55 kg (2.36-3.02 kg). Twelve patients (80%) were appropriate for gestational age, 3 (20%) were small for gestational age, and none were large for gestational age. All of the patients had moderate-to-severe or severe TR. Eleven patients (73%) had a lack of antegrade pulmonary artery blood flow, with anatomic pulmonary atresia in 8 (53%) and functional pulmonary atresia in 3 (20%). Thirteen patients (87%) were in a low cardiac output state preoperatively. Thirteen patients (87%) presented with at least mild RV dysfunction postnatally, among whom 11 (73%) and 3 (20%) needed preoperative inotropic support and ECMO, respectively. Thirteen patients (87%) were intubated preoperatively. Three patients (20%) had preoperative acute kidney injury.

**Table 1:** Baseline characteristics

Anatomy	All patients (n = 15)	Ebstein anomaly (n = 8)	Tricuspid valve dysplasia (n = 7)	*P* value
Male	8 (53%)	5 (62.5%)	3 (43%)	.62
Birth weight, kg	2.55 (2.36-3.02)	2.52 (2.38-2.92)	2.66 (2.22-3.11)	.77
Gestational age, wk	37 (36-39)	36 (35-39)	37 (37-39)	.86
Race/ethnicity				
White	8 (53%)	6 (75%)	2 (29%)	.13
Hispanic	8 (53%)	3 (37.5%)	5 (71%)	.31
Associated cardiac anomalies				
ASD/PFO	15 (100%)	8 (100%)	7 (100%)	
PDA	15 (100%)	8 (100%)	7 (100%)	
VSD	3 (20%)	1 (12.5%)	2 (29%)	
Anatomic pulmonary atresia	8 (53%)	3 (37.5%)	5 (71%)	
Pulmonary stenosis	1 (7%)	0 (0%)	1 (14%)	
Extracardiac malformations	2 (13%)	1 (12.5%)	1 (14%)	1.0
Genetic syndromes				.9
Down syndrome	2 (13%)	1 (12.5%)	1 (14%)	
CHARGE syndrome	1 (7%)	0 (0%)	1 (14%)	
Prenatal diagnosis	12 (80%)	6 (75%)	6 (86%)	1.0
Degree of TR				
Severe	11 (73%)	6 (75%)	5 (71%)	1.0
Moderate-severe	4 (27%)	2 (25%)	2 (29%)	1.0
TR jet velocity, m/s	2.8 (2.5-3.6)	2.6 (2.4-2.8)	3.3 (2.9-3.9)	.13
Lack of antegrade pulmonary artery flow	12 (80%)	7 (87.5%)	5 (71%)	.57
Anatomic pulmonary atresia	8 (53%)	3 (37.5%)	5 (71%)	.31
Functional pulmonary atresia	4 (27%)	4 (50%)	0 (0%)	.08
Prostaglandin administration	13 (87%)	6 (75%)	7 (100%)	.47
Inhaled nitric oxide	13 (87%)	7 (87.5%)	6 (86%)	1.0
Prenatal NSAIDs	2 (13%)	2 (25%)	0 (0%)	.47
Low cardiac output	13 (87%)	6 (75%)	7 (100%)	.47
Right ventricular dysfunction	13 (87%)	7 (87.5%)	6 (86%)	1.0
Left ventricular dysfunction	11 (73%)	5 (62.5%)	6 (86%)	.57
Preoperative inotrope support	11 (73%)	5 (62.5%)	6 (86%)	.57
Extracorporeal membrane oxidation	3 (20%)	1 (12.5%)	2 (29%)	.57
Preoperative lactate, mg/dL	2.60 (1.50-3.98)	1.64 (1.55-4.79)	2.70 (1.85-3.13)	1.0
Preoperative creatinine, mg/dL	0.76 (0.45-0.88)	0.82 (0.66-0.90)	0.47 (0.37-0.80)	.16
Acute kidney injury	3 (20%)	2 (25%)	1 (14%)	1.0
Noninvasive positive pressure ventilation	2 (13%)	2 (25%)	0 (0%)	.47
Intubated	13 (87%)	6 (75%)	7 (100%)	.47

Data are presented as number of patients (percent of total) or median (interquartile range). *P* values were calculated using Fisher exact test for categorical variables and the Wilcoxon rank sum test for continuous variables. *ASD*, Atrial septal defect; *PFO*, patent foramen ovale; *PDA*, patent ductus arteriosus; *VSD*, ventricular septal defect; *CHARGE*, coloboma, heart defects, atresia choanae, growth retardation, genital abnormalities, and ear abnormalities; *NSAIDs*, nonsteroidal anti-inflammatory drugs; *TR*, tricuspid regurgitation.

Of the 15 patients in this cohort, 8 (53%) were diagnosed with EA and 7 (47%) were diagnosed with TVD. All of the patients with FPA and circular shunt physiology had EA (4 vs 0, *P* = .08) and there was a trend toward a lower TR jet velocity in the patients with EA (2.6 vs 3.3 m/s, *P* = .13). Patient characteristics were otherwise similar between EA and TVD, with no significant difference in degree of TR, right or left ventricular function, or medical management. All patients with FPA and EA received a Starnes procedure. One patient with FPA was on ECMO preoperatively, and 2 patients with FPA had elevated postoperative lactate levels, both of whom died.

### Preoperative, Intraoperative, and Immediate Postoperative Characteristics Stratified by Surgery

Nine patients (60%) underwent the Starnes procedure, 5 (56%) for EA and 4 (44%) for TVD. Six patients (40%) underwent a TVr, 3 (50%) for EA and 3 (50%) for TVD. One patient who initially underwent a TVr but needed to be converted to a Starnes procedure during the index hospitalization for hemodynamic instability was discharged home alive and was analyzed as a survival case in the Starnes group. Demographic information stratified by surgical strategy is summarized in Table 2. The patients who underwent the Starnes procedure had a younger age at operation (2 vs 17 days, *P* = .05) and were more likely to have a lack of antegrade pulmonary blood flow (100% vs 50%, *P* = .04). The Starnes patients also tended to be in a low cardiac output state (100% vs 67%, *P* = .14), intubated (100% vs 67%, *P* = .14), and have a lower TR jet velocity (2.8 vs 3.9 m/s, *P* = .11).

**Table 2:** Preoperative, intraoperative, and postoperative characteristics stratified by type of surgery

Type of surgery	All patients (n = 15)	Starnes procedure (n = 9)	TV repair (n = 6)	*P* value
Male	8 (53%)	5 (56%)	3 (50%)	1.0
Birth weight, kg	2.55 (2.36-3.02)	2.66 (2.40-3.03)	2.54 (2.38-2.80)	.68
Gestational age (wk completed)	37 (36-39)	37 (35-39)	36 (36-38)	.63
Age at surgery, d	8 (2-21)	2 (2-10)	17 (8-47)	.05
Weight at surgery, kg	2.63 (2.21-3.05)	2.94 (2.30-3.10)	2.56 (2.21-2.76)	.60
Year of surgery				
2018-2023	9 (60%)	7 (78%)	2 (33%)	.14
2005-2017	6 (40%)	2 (22%)	4 (67%)	.14
Race/ethnicity				
White	8 (53%)	5 (56%)	3 (50%)	1.0
Hispanic	8 (53%)	4 (44%)	4 (67%)	.61
Associated cardiac anomalies				–
ASD/PFO	15 (100%)	9 (100%)	6 (100%)	
PDA	15 (100%)	9 (100%)	6 (100%)	
VSD	3 (20%)	1 (11%)	2 (33%)	
Anatomic pulmonary atresia	8 (53%)	5 (56%)	3 (50%)	
Pulmonary stenosis	1 (7%)	0 (0%)	1 (17%)	
Extracardiac malformations	2 (13%)	0 (0%)	2 (33%)	.14
Genetic syndromes				.21
Down syndrome	2 (13%)	0 (0%)	2 (33%)	
CHARGE syndrome	1 (7%)	0 (0%)	1 (17%)	
Prenatal diagnosis	12 (80%)	7 (78%)	5 (83%)	1.0
Diagnosis				
Ebstein anomaly	8 (53%)	5 (56%)	3 (50%)	1.0
Tricuspid valve dysplasia	7 (47%)	4 (44%)	3 (50%)	1.0
Degree of tricuspid regurgitation				
Severe	11 (73%)	8 (89%)	3 (50%)	.24
Moderate-severe	4 (27%)	1 (11%)	3 (50%)	.24
TR jet velocity, m/s	2.8 (2.5-3.6)	2.8 (2.4-3.0)	3.9 (2.8-4.2)	.11
Lack of antegrade pulmonary artery flow	12 (80%)	9 (100%)	3 (50%)	.04
Anatomic pulmonary atresia	8 (53%)	5 (56%)	3 (50%)	1.0
Functional pulmonary atresia	4 (27%)	4 (44%)	0 (0%)	.10
Prostaglandin administration	13 (87%)	8 (89%)	5 (83%)	1.0
Inhaled nitric oxide	13 (87%)	9 (100%)	4 (67%)	.14
Prenatal NSAIDs	2 (13%)	2 (22%)	0 (0%)	.49
Low cardiac output	13 (87%)	9 (100%)	4 (67%)	.14
Right ventricular dysfunction	13 (87%)	9 (100%)	4 (67%)	.14
Left ventricular dysfunction	11 (73%)	8 (89%)	4 (67%)	.24
Preoperative inotrope support	11 (73%)	8 (89%)	3 (50%)	.24
Extracorporeal membrane oxidation	3 (20%)	2 (22%)	1 (17%)	1.0
Preoperative lactate, mg/dL	2.60 (1.50-3.98)	3.26 (1.40-5.07)	2.10 (1.60-2.68)	.41
Preoperative creatinine, mg/dL	0.76 (0.45-0.88)	0.80 (0.61-0.88)	0.53 (0.30-0.78)	.19
Acute kidney injury	3 (20%)	3 (33%)	0 (0%)	.23
Noninvasive positive pressure ventilation	2 (13%)	0 (0%)	2 (33%)	.14
Intubated	13 (87%)	9 (100%)	4 (67%)	.14
CBP time, min	127 (111-137)	127 (108-143)	129 (117-131)	1.0
Crossclamp time, min	54 (36-71)	38 (25-54)	71 (63-78)	.05
Postoperative ECMO	1 (7%)	0 (0%)	1 (17%)	.4
Time to extubation, d	4 (4-7)	5 (4-8)	4 (4-4)	.51
Reintubations	5 (33%)	4 (44%)	1 (17%)	.58
Time to enteral feeding, d	7 (6-11)	8 (5-12)	6 (6-7)	.47
Delayed sternal closure	8 (53%)	6 (67%)	2 (33%)	.31
Heart block	3 (20%)	2 (22%)	1 (17%)	1.0
Unplanned reoperation	3 (20%)	3 (33%)	0 (0%)	.23
POD 1 lactate, mg/dL	2.00 (1.50-5.16)	2.03 (1.50-6.51)	1.90 (1.58-2.14)	.95
POD 1 creatinine, mg/dL	0.54 (0.45-0.65)	0.56 (0.49-0.63)	0.47 (0.38-0.64)	.32

Data are presented as number of patients (percent of total) or median (interquartile range). *P* values were calculated using the Fisher exact test for categorical variables and the Wilcoxon rank sum test for continuous variables. *TV*, Tricuspid valve; *ASD*, atrial septal defect; *PFO*, patent foramen ovale; *PDA*, patent ductus arteriosus; *VSD*, ventricular septal defect; *CHARGE*, coloboma, heart defects, atresia choanae, growth retardation, genital abnormalities, and ear abnormalities; *NSAIDs*, nonsteroidal anti-inflammatory drugs; *CBP*, cardiopulmonary bypass; *ECMO*, extracorporeal membrane oxidation; *POD*, postoperative day.

Types of surgical procedures, intraoperative characteristics, and immediate postoperative outcomes are summarized in Tables 2 and 3. Crossclamp times in patients who underwent Starnes were shorter (38 minutes vs 71 minutes, *P* = .05). Starnes surgery tended to be performed in the more recent era, with more Starnes procedures than TVr performed in 2018 to 2023 (7 vs 2, *P* = .14) and more TVr than Starnes performed in 2005 to 2017 (4 vs 2, *P* = .14).

**Table 3:** Procedures performed

Type of TV surgery	Starnes (n = 9)	TVr (n = 6)
PDA ligation	9 (100%)	6 (100%)
Reduction atrioplasty	4 (44%)	6 (100%)
Blalock–Taussig–Thomas shunt or central shunt	9 (100%)	1 (17%)
ASD management		
Atrial septectomy	9 (100%)	
Partial closure		3 (50%)
RVOT management		
PA ligation or division	6 (67%)	0 (0%)
PV transannular patch	0 (0%)	3 (50%)
RV-to-PA conduit	0 (0%)	1 (17%)
PV repair	0 (0%)	1 (17%)
VSD closure	0 (0%)	1 (17%)
ECMO decannulation	2 (22%)	0 (0%)
TVr procedures (n = 6)		
Annuloplasty alone		2 (33%)
Commissuroplasty alone		1 (17%)
Division of restrictive chordae and commissuroplasty		1 (17%)
Annuloplasty and Sebening stitch		1 (17%)
Cone repair		1 (17%)

Data are presented as number of patients (percent of total) or median (interquartile range). *TV*, Tricuspid valve; *PDA*, patent ductus arteriosus; *ASD*, atrial septal defect; *RVOT*, right ventricular outflow tract; *PA* pulmonary artery; *PV*, pulmonary valve; *RV*, right ventricle; *VSD*, ventricular septal defect; *ECMO*, extracorporeal membrane oxidation; *TVr*, tricuspid valve repair.

### Outcomes

#### Immediate outcomes and operative mortality

The most frequent complications were heart block (3 patients, 20%) and unplanned reoperation (3 patients, 20%). The reasons for reoperation were epicardial pacemaker placement, hemopericardium, and revision of Blalock–Taussing–Thomas shunt. One patient (7%) required ECMO postoperatively. Median (IQR) length of stay was 56 days (36-76 days).

**Figure 1:**
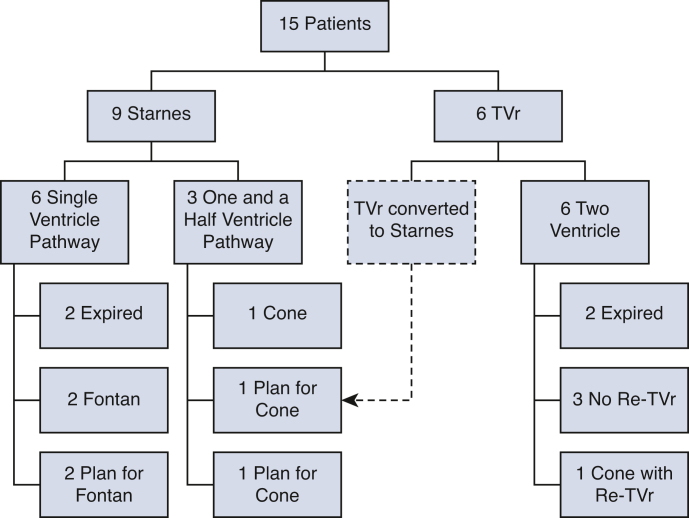
Flow diagram of patient outcomes. The *dashed box/line* indicates 1 patient who initially underwent a TVr at 5 days of age but was converted to a Starnes procedure at 33 days of age during the same hospitalization due to hemodynamic instability. *TVr*, Tricuspid valve repair.

A figure showing patient surgery and outcomes is presented in Figure 1. Individual patient outcomes are tabulated in Table 4. Operative mortality was 20% (n = 3) (95% confidence interval [CI], 0%-39%). Two had a TVr and one had a Starnes. Two patients died of refractory low cardiac output syndrome complicated with multiorgan failure (one Starnes for EA and one TVr for EA) at day 1 and 3 postoperatively, and the third patient died of recurrent ventricular tachycardia on postoperative day 40 after a TVr for TVD. None of these patients had a reoperation. When comparing the eras (2005-2017 vs 2018-2023), there was no difference in mortality (*P* = .81) or reintervention on the TV (*P* = .23).

**Table 4:** Individual patient diagnoses, management, and outcomes

Patient number	Year of birth	Diagnosis	PV flow status	Type of surgery	TV reintervention	Ventricular status	Mortality
1	2006	EA	Antegrade	TVr	No	2	No
2	2009	EA	FPA	Starnes	No	1	Yes
3	2015	EA	FPA	Starnes	No	1	No
4	2018	EA	FPA	Starnes	No	1	Yes
5	2018	EA	APA	TVr	Yes	2	No
6	2005	EA	APA	TVr	No	2	Yes
7	2021	EA	FPA	Starnes	Yes	1.5	No
8	2023	EA	APA	Starnes	No	1	No
9	2022	TVD	APA	Starnes	No	1	No
10	2022	TVD	APA	Starnes	No	1	No
11	2018	TVD	APA	Starnes	No	1	No
12	2012	TVD	Antegrade	TVr	No	2	No
13	2021	TVD	Antegrade	TVr	No	2	Yes
14	2014	TVD	APA	TVr	No	2	No
15	2021	TVD	APA	Starnes	Yes	1	No

*PV*, Pulmonary valve; *TV*, tricuspid valve; *EA*, Ebstein anomaly; *TVr*, tricuspid valve repair; *FPA*, functional pulmonary atresia; *APA*, anatomic pulmonary atresia; *TVD*, tricuspid valve dysplasia; *TVD*, tricuspid valve dysplasia.

#### Mortality at last follow-up

**Figure 2:**
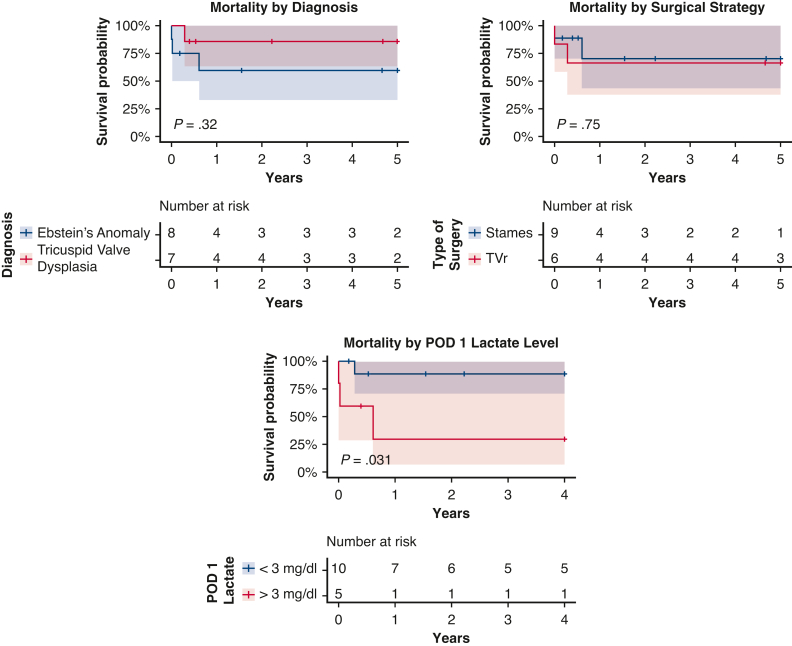
Kaplan–Meier plots of mortality stratified by diagnosis, type of surgery performed, and POD 1 lactate level. Log rank test used to calculate *P* values. *TVr*, Tricuspid valve repair; *POD*, postoperative day.

**Figure 3:**
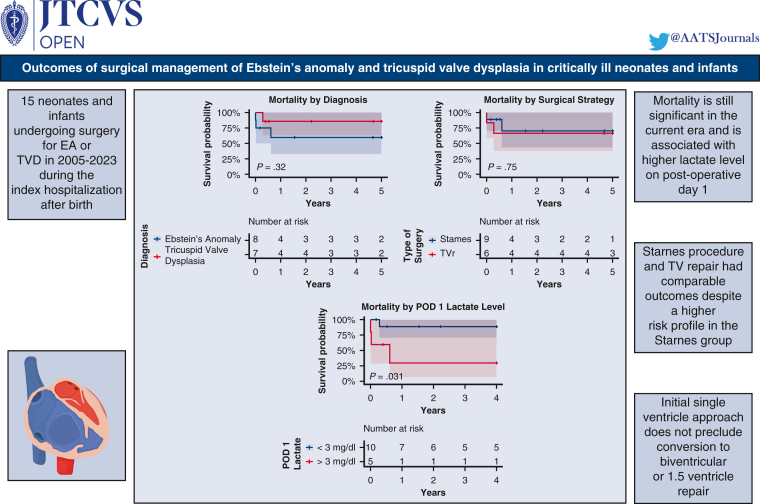
Graphical abstract illustrating the main implications of the study. *EA*, Ebstein anomaly; *TVD*, tricuspid valve dysplasia; *TVr*, tricuspid valve repair; *POD*, postoperative day; *TV*, tricuspid valve.

The patients were followed for a median of 1.55 years (0.35-6.21 years). In addition to the 3 patients who died during the initial hospital admission, 1 additional patient who had a Starnes for EA passed away at 8 months of age at home without a clear etiology. A total of 4 of 15 patients died during the study period, 3 with EA and 1 with TVD. Mortality at last follow-up was 27% (95% CI, 4%-48%) overall, 37.5% (95% CI, 0%-63.5%) for EA and 14% (95% CI, 0%-42%) for TVD. This difference was not significant (*P* = .32). The mortality rate for patients who underwent the Starnes procedure was 22% (95% CI, 0%-48%) and for patients who underwent TVr was 33% (95% CI, 0%-62%). This difference was also not significant (*P* = .75). Kaplan–Meier survival analyses are shown in Figures 2 and 3.

On univariable Cox proportional hazards analysis of risk factors for mortality, postoperative day 1 lactate was significantly associated with a greater risk of mortality (hazard ratio, 1.45; *P* = .01). The Cox analysis results are shown in Table 5.

**Table 5:** Cox proportional hazards analysis of factors associated with mortality

Risk factor	Hazard ratio	95% confidence interval	*P* value
POD 1 lactate	1.45	1.08-1.95	.01
Reintubation	2.70	0.35-20.86	.34
Lack of antegrade pulmonary artery flow	0.85	0.09-8.23	.89
Anatomic pulmonary atresia	0.31	0.03-3.05	.32
Functional pulmonary atresia (with circular shunt)	2.79	0.39-20.04	.31
Preoperative creatinine	2.14	0.06-77.31	.68
Preoperative lactate	1.18	0.83-1.68	.36
Preterm birth	3.47	0.36-33.68	.28
Severe TR	1.09	0.11- 10.57	.94
TR jet velocity	0.75	0.21-2.72	.66
Age at operation	1.01	0.97-1.06	.59
Postoperative heart block	1.49	0.15-14.48	.73
Ebstein anomaly	3.02	0.31-29.32	.34
Tricuspid valve dysplasia	0.33	0.03-3.22	.34
Starnes procedure	0.73	0.10-5.20	.75
Tricuspid valve repair	1.37	0.19-9.72	.75

For continuous variables, hazard ratio refers to the change in proportional hazard with each standard deviation. For categorical variables, hazard ratio refers to the change in proportional hazard when the factor is present. *POD*, Postoperative day; *TR*, tricuspid regurgitation.

#### Ventricular status

Among the 9 patients who underwent the Starnes procedure, 6 remained on the single-ventricle pathway. Three patients in the Starnes group are on the 1.5- or 2-ventricle conversion pathway: one had a Glenn then was converted to a cone repair after 1 year of age; the 2 others are awaiting conversion to a cone repair.

Among the patients who underwent TVr, 6 remain with a biventricular repair and 1 was converted to a Starnes during the index hospitalization. One patient underwent a cone procedure as a neonate and subsequently required a reintervention on his TV.

#### Tricuspid valve regurgitation

At last follow-up, among survivors who did not get their TV excluded (n = 4), 1 patient (25%) had mild TR, 2 patients (50%) had moderate TR (33%), and 1 patient (25%) had moderate-to-severe TR.

## Discussion

Patients with EA and TVD requiring surgery as neonates and infants represent a rare and challenging cohort of patients. As yet, the optimal approach remains unclear, with some groups strongly advocating primary biventricular repair,^6^ whereas others suggest that initial Starnes procedure is a more reproducible approach.^1^ A 2018 study on surgery for neonatal and infant EA from the STS database demonstrated that an initial biventricular repair was attempted in 40% of neonates, Starnes procedure was performed in 9%, whereas other palliative procedures were performed in the remaining patients.^10^ This demonstrates considerable variability in practice. In that study, the overall mortality rate was 18.6%; 25% among patients who underwent TV exclusion, and 28% among patients who had a primary biventricular repair.^10^

In the present study, we analyzed the outcomes of critically ill neonates and infants who underwent surgery for EA and TVD at a single center from 2005 to 2023 during their index hospitalization after birth. The overall mortality rate in this cohort was 27%, which is similar to the published mortality rate for neonates and infants with EA and TVD despite excluding the noncritically ill patients who were discharged home before surgery. The published mortality rate of neonates and infants who undergo surgery for EA or TVD ranges from 18.6% to 53%.^10^, ^11^, ^12^, ^13^

In our cohort, the majority of the deaths (3 of 4) were in patients with EA, whereas only 1 patient with TVD died. Interestingly, all of the patients who had FPA with circular shunt physiology had EA. In addition, patients with EA had a trend toward a lower TR jet velocity compared with patients who underwent TVr, which is a known risk factor for adverse outcomes.^11^ These 2 factors, in addition to the known RV myocardial abnormality in patients with EA, may explain this greater number of deaths in the EA group compared with the TVD group.

On Cox regression modeling, postoperative day 1 lactate was associated with mortality. This finding may reflect the poor hemodynamic status of these patients perioperatively. Factors previously identified in the literature as being associated with mortality include a lack of antegrade pulmonary blood flow and TR jet velocity <2.5 m/s^11^; however, they were not correlated with mortality in this cohort.

The most common complication identified in this cohort was complete heart block in 3 of 15 patients (20%). The risk to the conduction system in TV procedures is well recognized, but the incidence of this complication is high even despite the known risk. The patients who had this complication underwent a Starnes procedure and/or an annuloplasty. In order to reduce the risk of complete heart block in the future, we have adopted a strategy of placing sutures at the junction between the valve tissue and the annulus rather than the annulus itself with shallow bites with 6-0 or 7-0 PROLENE sutures.

The patients who underwent the Starnes procedure had a worse preoperative risk profile, including a younger age at operation, greater incidence of lack of antegrade pulmonary blood flow, and a trend toward intubation, being in a low cardiac output state, and lower TR jet velocity. A greater mortality would be expected in these patients who underwent Starnes, and the lack of this finding would seem to indicate that the Starnes procedure had a protective effect when offered to those patients at greater risk profile.^1^ In these patients, the shorter crossclamp time and greater reproducibility of the Starnes procedure may be beneficial. Indeed, it does not rely on a technically challenging repair of the tricuspid valve or on the function of an overloaded and often dysfunctional right ventricle. Interestingly, there was a trend toward more Starnes procedures performed after 2017, which may reflect a shift in our surgical practice toward preferring the Starnes procedure for critically ill neonates who require surgery before discharge from the neonatal intensive care unit.

The mortality rate in neonates and infants who underwent TVr in our cohort was similar to the patients who underwent Starnes despite having a more favorable risk profile. Multiple approaches to TVr in this population are in use, including a monocuspid repair as described by Knott-Craig and Boston,^13^ a cone repair,^4^ and other techniques including annuloplasty and commissuroplasty that were employed for many of the patients in this cohort. There are very few studies of neonates and infants undergoing the cone repair.^7^^,^^8^ In our cohort, one neonate underwent a cone repair at 9 days of age and survived.

One patient was converted from a Starnes to a cone repair after having a Glenn, thus transitioning from a 1-ventricle pathway toward a 1.5-ventricle repair. Two other patients who underwent Starnes are awaiting a Starnes take-down and a conversion to a 1.5- or 2-ventricle repair. These patients add to the growing body of literature^14^^,^^15^ reporting that the Starnes procedure can be a first step in the neonatal period before conversion to a tricuspid valve repair and a 2- or 1.5-ventricle physiology at a more favorable time. This staged strategy (Starnes followed by 1.5-ventricle conversion) may bring a survival advantage in the neonatal period without the commitment to lifelong single-ventricle physiology. In light of this evolving surgical strategy, we have altered our operative technique to use polytetrafluoroethylene rather than autologous pericardium as the patch material for RV exclusion in the Starnes procedure to limit scar tissue formation and facilitate taking down the patch.

Based on these results, we can suggest a strategy to approach surgical management for EA and TVD in the neonate and infant. For the youngest and sickest patients as well as those with unfavorable tricuspid anatomy, a Starnes procedure seems to provide the safest and most reproducible option. The Starnes procedure may allow for the most reliable outcomes in the neonatal/infant period, with increasing evidence that this does not preclude later biventricular or 1.5-ventricle repair. Indeed, once beyond the neonatal phase, conversion to biventricular repair should be considered before proceeding further down the single ventricle pathway. Conversely, in more stable patients with favorable anatomy, proceeding directly to a biventricular repair, with consideration for a TV repair (cone repair or monocuspid-based repair for EA, or the croissant repair for TVD^16^), appears reasonable. Such a strategy may allow for the greatest number of patients to achieve biventricular repair while avoiding excess mortality in the neonatal period and infancy.

### Limitations

This study is limited by its small sample size and retrospective nature. In particular, the analysis of factors associated with mortality and survival analysis in general were limited by the small number of events. In addition, the time period of the study reflects a changing surgical strategy. In particular, the Starnes procedure became more prevalent towards the end of the study period. The approach of the individual surgeons was also not standardized and influenced the surgical strategy applied for each patient.

## Conclusions

Surgery for neonatal and infant EA and TVD is still associated with significant mortality in the current era. The risk of mortality seems to be associated with a greater lactate level on postoperative day 1. The Starnes procedure and neonatal TVr seem to have comparable outcomes despite a greater-risk profile in the Starnes group. An initial single-ventricle approach does not preclude conversion to biventricular repair.

### Webcast

You can watch a Webcast of this AATS meeting presentation by going to: https://www.aats.org/resources/outcomes-of-surgical-management-of-ebstein-anomaly-in-neonates-and-infants.

**Figure:**
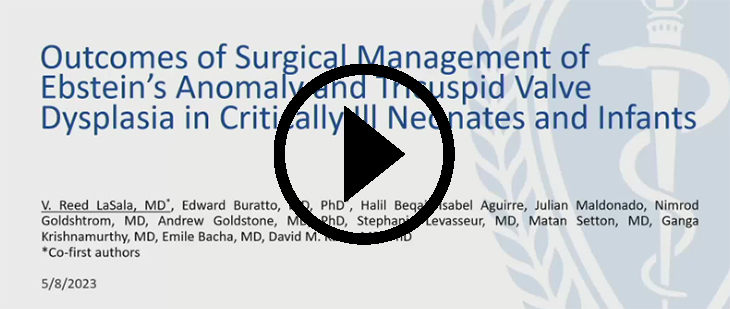

## Conflict of Interest Statement

The authors reported no conflicts of interest.

The *Journal* policy requires editors and reviewers to disclose conflicts of interest and to decline handling or reviewing manuscripts for which they may have a conflict of interest. The editors and reviewers of this article have no conflicts of interest.
